# bCNN-Methylpred: Feature-Based Prediction of RNA Sequence Modification Using Branch Convolutional Neural Network †

**DOI:** 10.3390/genes12081155

**Published:** 2021-07-28

**Authors:** Naeem Islam, Jaebyung Park

**Affiliations:** 1 Core Research Institute of Intelligent Robots, Jeonbuk National University, Jeonju 54896, Korea; naeem@jbnu.ac.kr; 2 College of Electrical & Mechanical Engineering, NUST, Islamabad 44000, Pakistan; 3 Division of Electronics and Information Engineering, Jeonbuk National University, Jeonju 54896, Korea

**Keywords:** RNA modification, branch convolutional neural network, N^6^-methyladenosine, circular encoding

## Abstract

RNA modification is vital to various cellular and biological processes. Among the existing RNA modifications, N^6^-methyladenosine (m6A) is considered the most important modification owing to its involvement in many biological processes. The prediction of m6A sites is crucial because it can provide a better understanding of their functional mechanisms. In this regard, although experimental methods are useful, they are time consuming. Previously, researchers have attempted to predict m6A sites using computational methods to overcome the limitations of experimental methods. Some of these approaches are based on classical machine-learning techniques that rely on handcrafted features and require domain knowledge, whereas other methods are based on deep learning. However, both methods lack robustness and yield low accuracy. Hence, we develop a branch-based convolutional neural network and a novel RNA sequence representation. The proposed network automatically extracts features from each branch of the designated inputs. Subsequently, these features are concatenated in the feature space to predict the m6A sites. Finally, we conduct experiments using four different species. The proposed approach outperforms existing state-of-the-art methods, achieving accuracies of 94.91%, 94.28%, 88.46%, and 94.8% for *the H. sapiens*, *M. musculus*, *S. cerevisiae*, and *A. thaliana* datasets, respectively.

## 1. Introduction

Gene expression involves the utilization of gene information to synthesize functional gene products. It is a multilayered process that starts by controlling the information of a particular sequence encoded in DNA, which is then copied to RNA molecules. Subsequently, the RNA molecules branch off to transfer their sequence information into polypeptides (coding RNAs, mRNAs) or non-coding RNAs. More importantly, RNA functionality relies not only on the sequence information, but also on splicing; in particular, alternative splicing can diversify RNAs. Every RNA nucleotide can be chemically modified or interchanged (RNA edited). More than 150 RNA modifications have been documented since the discovery of the first type of RNA modification in 1957. Among these modifications, $N^{6}$ methyladenosine (m6A) is the most abundant and typical modification that exists in various species. It is assumed to be associated closely with diverse biological processes, including RNA localization and degradation [1], RNA structural dynamics [2], alternative splicing [3], primary microRNA processing [4], cell differentiation and reprogramming [5], and regulation of the circadian clock [6]. Recent research has shown that RNA modifications are associated with different diseases, including metabolic disorders, neurological disorders, cancer, and cardiovascular diseases. For instance, m6A is associated with cancer, obesity [7], acute myelogenous leukemia [8], the Zika virus [9], and depressive disorders [10]. Apart from m6A, m1A is associated with X-linked intractable epilepsy, multiple respiratory chain deficiencies [11], and neurodevelopmental regression [12]. A-to-I is associated with cancer [13,14] and neurological disorders [15]. m5C is associated with breast cancer [16] and intellectual disability syndrome [17,18]. Further details regarding the associations between RNA modification and diseases are available in [19,20].

## 2. Related Studies

Considering the importance of RNA modification, particularly m6A, site identification is required for a better understanding of their functional mechanisms. High-throughput experimental techniques, such as MERIP and m6A-seq, have been proposed in [21,22], respectively. MERIP [21] identified the mRNAs of mammalian genes that contain m6A, indicating that m6A is a typical base modification of mRNA. It was observed that m6A sites enriched near stop codons and in three prime untranslated regions. m6A-seq [22] provides *mouse* and *human* m6A modification landscapes in a transcriptome-wide manner based on massively parallel sequencing and antibody-mediated captures. It was reported that m6A sites appeared around stop codons and within long internal exons. However, these approaches present the following limitations: (1) low accuracy in locating the correct positions of m6A sites, (2) high computational complexity, and (3) limited applicability of large-scale identification of m6A sites. Therefore, accurate and fast methods are required for the correct identification of m6A sites [23].

Recently, deep-learning (DL)- and machine-learning (ML)-based modification prediction approaches have been developed to identify m6A sites. A pioneering study that used the ML approach was reported in [24], where “iRNA-Methyl” was proposed for m6A site identification. In that approach, the authors exploited the “pseudo dinucleotide composition,” into which three RNA physiochemical properties were incorporated, while formulating the RNA sequences. To develop their m6A site predictor model, they used a support vector machine. In [25], the authors proposed pRNAm-PC, in which RNA sequence samples were expressed by incorporating an additional mode of pseudo dinucleotide composition, whose components were derived from a physical–chemical matrix via a series of auto-covariance and cross-covariance transformations. In [26], the authors proposed a bioinformatics model known as RNA-MethylPred. They developed their model by incorporating bi-profile Bayes, dinucleotide composition, and k-nearest neighbor scores for three feature extractions, yielding improved results compared with previous approaches that used only a single feature descriptor. In [27], the authors showed that using a combination of the binary encoding scheme and k-mer frequency resulted in improved performance. In [28], the authors proposed a powerful prediction tool named “SRAMP.” In their proposed approach, multiple types of feature descriptors were used, including a positional binary encoding of nucleotide sequence, k-nearest neighbor encoding, nucleotide pair spectrum encoding, and secondary structure pattern for training the ensemble predictive model based on the random forest for m6A site identification. Their proposed approach achieved relatively better performances compared with other existing predictors. A new m6A site predictor named “RNAMethyPre” was proposed in [29], where compositional information and position-specific information were used to develop predictive models for predicting m6A sites in both *human and mouse* species. A DL-based algorithm was proposed in [30] to generate latent features to improve predictive performance. In [31], the authors proposed a sequence-based predictor for detecting m6A sites in RNA sequences for multiple species. They proposed a feature representation algorithm by encoding sequences with dinucleotide binary encoding and local position-specific dinucleotide frequency. They combined an F-score algorithm with sequential forward search [32,33,34] to optimize the feature space and improve the representation capabilities. They applied the XGBoost algorithm to perform model training using available optimal features. Recently, Nazari et al. proposed a DL-based convolutional neural network (CNN), which they named iN6-Methyl (five step) [35], for m6A site prediction for benchmark species of *H. sapiens*, *M. musculus*, and *S. cerevisiae*. In their proposed approach, they extracted features using a natural-language-processing-based word2vec model. In this approach, each sequence was manually segmented into words with a length of k using a k-mer technique. They set the value of k to 3, and each word was mapped to its corresponding feature representation. As their model uses an entire genome for training, its computational complexity is high, whereas its prediction of m6A sites is slow. More recently, the authors of [36] proposed a CNN-based architecture for identifying m6A sites in RNA sequences, which they named pm6A-CNN. They used a combination of one-hot encoding and nucleotide chemical properties (NCP) as an input to the model. Furthermore, they used a grid search algorithm to determine the optimal parameters of their model. Their proposed approach achieved improved performance compared with existing approaches. However, the combination of features as an input to the model presents limitations in terms of training if the feature representations are highly nonlinear.

The main contributions of this paper are as follows:

(1). bCNN-Methylpred, a novel branch CNN: This network combines the features from different encoding schemes and accurately predicts m6A sites in different RNA sequences.
(2). A novel encoding scheme: We propose a novel circular encoding scheme that considers every possible combination of the four nucleotide bases in the RNA sequence, namely, adenine (A), cytosine (C), guanine (G), and uracil (U). The proposed encoding schemes further improves the accuracy of predicting m6A sites in different RNA sequences.
(3). Feature fusion: The proposed approach uses the individual encoded RNA sequences using three encoding schemes and then combines their features. Subsequently, the combined features are used to identify m6A sites in different RNA sequences.
(4). Biological interpretation of the proposed model: We investigate the proposed model from a biological perspective by interpreting the trained model based on a well-established interpretation procedure, i.e., in silico mutagenesis.

## 3. Materials and Methods

This section presents the different encoding schemes, proposed scheme, benchmark datasets, performance evaluation, discussion, and biological interpretation of the model.

### 3.1. Encoding Schemes

**Figure 1:**
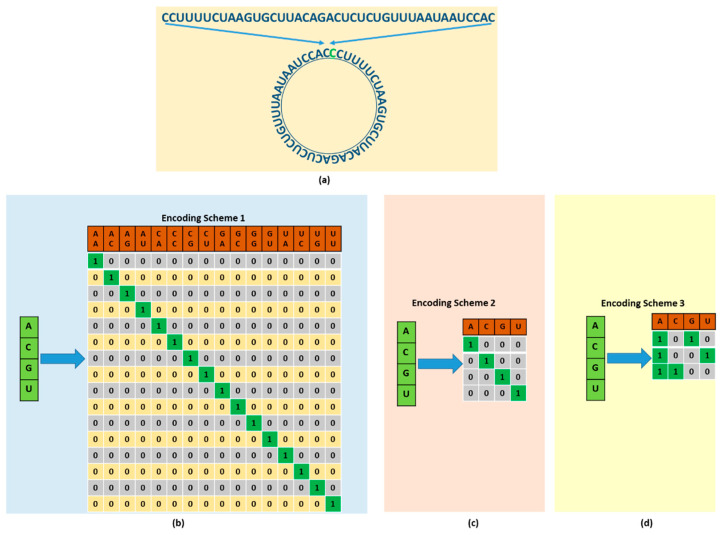
Representation of RNA sequences using different encoding schemes as input to the proposed approach. (**a**) shows the circular representation of RNA sequence. (**b**) shows circular encoding considering every possible combination of four nucleotides bases, and four nucleotides bases yield 16 possible configurations, as shown. (**c**) shows one-hot encoding. (**d**) shows the encoding of input sequence based on nucleotide chemical properties.

Representing the RNA sequence in a form that is acceptable for a deep neural network is the most basic and important step. Hence, we used a novel circular encoding scheme to represent the RNA sequence in addition to two typically used encoding techniques, i.e., one-hot encoding and NCP. The encoding schemes are shown in Figure 1. The proposed circular encoding scheme is based on a pairwise combination of the four nucleotide bases in the RNA sequences. First, we converted the sequence to a circular shape, as shown in Figure 1a; subsequently, we encoded it in a pairwise combination. We have a sequence of nucleotide bases A, C, G, and U of a specific length. To make pairs of the nucleotide bases, we start from the left and pick bases and make pairs. To make this combination circular, we replicate the first nucleotide base at the end of the sequence and make pairs of the modified sequence. Once the pairs are formed, we assign unique codes to each of these pairs. This encoding scheme considers every possible combination of nucleotide base pairs, resulting in 16 possible configurations. The corresponding bits of each pair were set to 1, whereas the remaining bits were set to zero. The overall configuration of the circular encoding is shown in Figure 1b. As the circular encoding considers every possible configuration and arrangement of the nucleotide bases in the RNA sequences, the model can be trained more effectively using more information. Consequently, the model will yield more accurate results in predicting modification sites in the RNA sequences. The advantages of including circular encoding are discussed and evaluated comprehensively in the performance evaluation section below. One-hot encoding is a binary representation of the four nucleotide bases, A, C, G, and U, in the RNA sequence, where nucleotide bases A, C, G, and U are represented as (1,0,0,0), (0,1,0,0), (0,0,1,0), and (0,0,0,1), respectively. A detailed description of one-hot encoding is presented in Figure 1c. The NCP is the representation of each nucleotide base in the RNA sequence based on three chemical groups in the three-dimensional Cartesian coordinate system. Each of the four nucleotide bases A, C, G, and U in the RNA sequences have different chemical properties. Considering the ring structures, A and G are purines comprising two rings. C and U are pyrimidines comprising one ring. Considering the formation of secondary structures, the hydrogen bond between A and U is weak, whereas the hydrogen bond between C and G is strong. Similarly, regarding the chemical functionality, A and C belong to the amino group, whereas G and U belong to the keto group. Considering these three chemical properties, the four nucleotide bases of the RNA sequences can be categorized into three distinct groups in the Cartesian coordinate system by assigning a value of 1 or 0. If the x-, y-, and z-coordinates represent the ring structure, hydrogen bond, and chemical functionality, respectively, then each nucleotide base can be encoded by ($x_{i}$, $y_{i}$, $z_{i}$), as shown in the figure below.

(1) $$x_{i}=\{\begin{array}{c} 1\;\;\;\;\;\;\;\;\;\;\;\;\;\;if\;s_{i}\in \;\left\{A,\;G\right\}\\ 0\;\;\;\;\;\;\;\;\;\;\;\;\;\;if\;s_{i}\in \;\left\{C,\;U\right\} \end{array}$$

(2) $$y_{i}=\{\begin{array}{c} 1\;\;\;\;\;\;\;\;\;\;\;\;\;\;if\;s_{i}\in \;\left\{A,\;U\right\}\\ 0\;\;\;\;\;\;\;\;\;\;\;\;\;\;if\;s_{i}\in \;\left\{C,\;G\right\} \end{array}$$

(3) $$z_{i}=\{\begin{array}{c} 1\;\;\;\;\;\;\;\;\;\;\;\;\;\;if\;s_{i}\in \;\left\{A,\;C\right\}\\ 0\;\;\;\;\;\;\;\;\;\;\;\;\;\;if\;s_{i}\in \;\left\{G,\;U\right\}, \end{array}$$

where $s_{i}$ represents the nucleotide bases. As shown in the configuration above, A is represented by (1, 1, 1), C by (0, 0, 1), G by (1, 0, 0), and U by (0, 1, 0). A detailed description of the NCP is presented in Figure 1d.

#### 3.1.1. Algorithm of Circular Encoding

1. Select a Sequence;
2. Replicate the first nucleotide base at the end of the sequence;
3. Make pairs of the nucleotides bases in the given sequence;
4. Assign unique binary code to each pair (Code assignment is shown in Figure 1b);
5. The generated code is used as input to the network.

#### 3.1.2. Example of Circular Encoding

1. Given a sequence as:
  ′CCUUUUCUAAGUGCUUACAGACUCUCUGUUUAAUAAUCCAU′;
2. Replicate the first base at the end;
3. The modified sequence is:
  ′CCUUUUCUAAGUGCUUACAGACUCUCUGUUUAAUAAUCCAUC′;
4. Make pairs of bases:
  ′(CC), (UU), (UU), (CU), (AA), (GU), (GC), (UU), (AC), (AG), (AC), (UC), (UC), (UG), (UU), (UA), (AU), (AA), (UC), (CA), (UC)′;
5. Assign binary code to each pair as shown in Figure 1b.

### 3.2. Proposed Approach

**Figure 2:**
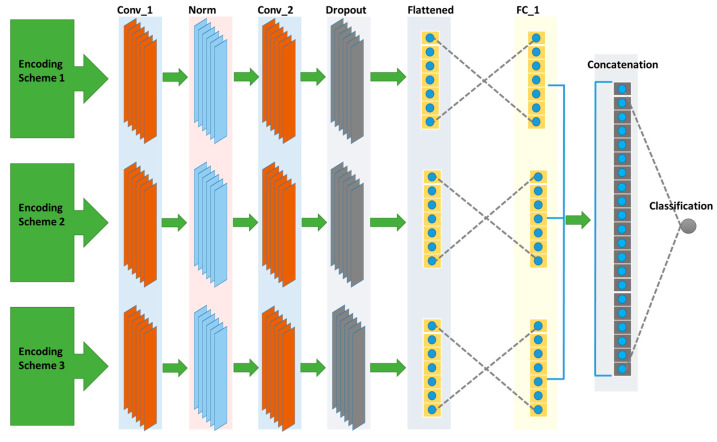
Branch convolutional neural network for identifying modification sites in different RNA sequences. The top branch uses the RNA sequence represented by the circular encoding scheme as input. The second and third branches use RNA sequence represented by one-hot encoding and NCP encoding schemes, respectively, as inputs. Each network branch comprises two convolution layers, one normalization layer, and two fully connected layers. The last fully connected layer uses concatenated features from three branches and predicts modification sites using a single output unit.

As a solution to the problems discussed above, we propose bCNN-Methylpred. The proposed network comprises three projection branches as shown in Figure 2. The first branch uses circular encodings as an input and projects them to the corresponding features. The second branch uses one-hot encoding as the input and generates the features in the feature space. Similarly, the third branch uses the NCP as the input and projects them to the required features in the feature space. Subsequently, the features generated by all branches of the proposed network are concatenated into a single feature space, which is then classified into positive and negative sequences in the last fully connected layer. The positive and negative sequences show the presence and absence of mA6 modification sites, respectively. Concatenating the features rather than the direct inputs is advantageous as the feature space is linearly separable compared with the nonlinear and non-separable input space. To further elaborate this, the RNA sequence is first encoded using the three encoding schemes including one-hot encoding, circular encoding, and nucleotide chemical properties encoding. Once the encoding is complete, the next step is to use this encoded sequence in the network and predict the modification sites. The previous approaches first combine the encoded sequences and then provide the combined sequence as input in their network to predict the modification sites. This combination is linear, i.e., the encoded sequence is not passed through a non-linear function while in our approach, we first pass the individual encoded sequence through a neural network. Note that the neural networks with multiple layers and activation functions act as nonlinear function approximations. The nonlinear outputs of each branch are then combined and provided as input to the last fully connected layer for predicting the modification sites in the given RNA sequence. Considering the proposed network configuration, each branch contains two convolutional layers, one group normalization layer, one dropout layer, one fully connected layer, and an output classification layer. The input to the first, second, and third branches of bCNN-Methylpred are a 16-bit vector, 4-bit vector, and 3-bit vector, respectively. The first convolution layer of each branch in the proposed network comprises 32 filters, each of which has a size of 5. The output of the first convolution layer is passed through the ReLU activation function. The output of the activation function is passed through the group normalization layer [37] with a group size of 4. The second convolution layer uses the output of group normalization as the input. This convolution layer has 16 filters with a filter size of 3. The output of the second convolution layer is passed through the ReLU activation function, which is then flattened into a one-dimensional feature vector. Subsequently, the one-dimensional feature vector is passed through a dropout layer with a dropout rate of 0.5. The output of the dropout layer is passed through the first fully connected layer with 24 features. The first fully connected layer of each branch is concatenated to form 72 hidden features. Subsequently, these hidden units are connected to the classification layer. The classification layer has a single output neuron to perform a binary classification that determines whether modification has occurred in the RNA sequence. In other words, it indicates the presence or absence of m6A sites in the RNA sequence. We used a nonlinear sigmoid activation function for the classification layer output. Additionally, we used l2 regularization to avoid overfitting of the network. To train the parameters of the network, we used the Adam optimizer with a learning rate of 0.001. We set the batch size to 32, and early stopping based on validation loss was utilized for the maximum number of training iterations, i.e., 1000. The bCNN-Methylpred network was implemented using Keras, an open-source DL library, on a GPU-based PC comprising an Intel (R) Core i9-9940X CPU, a 132.0 GB RAM, and four NVIDIA GeForce RTX 2080 Ti graphics cards. The bCNN-Methylpred network is lightweight and can be operated on any platform without using a GPU. Moreover, it requires an extremely small memory capacity of 284.2 kB. Hence, the proposed network is not affected by computational complexity and memory. Although we used four NVIDIA GeForce RTX 2080 Ti graphics cards, the sole purpose was to operate multiple networks simultaneously for training using different species. Further details regarding hyperparameter optimization are shown in Table 1.

#### Network Architecture and Training

The stepwise training algorithm of the proposed bCNN-Methylpred network is explained below. In the first step, we obtained the input representations of the four nucleotide bases of the RNA sequences using circular encoding, one-hot encoding, and NCP schemes. In the second step, the generated input representation from the circular encoding, one-hot encoding, and NCP are projected onto the corresponding feature representation using the first, second, and third branches of the network as follows:

(4) $$z_{1}=f1\left(seq_{1}\right)$$

(5) $$z_{2}=f2\left(seq_{2}\right)$$

(6) $$z_{3}=f3\left(seq_{3}\right),$$

where $z_{1}$, $z_{2}$, and $z_{3}$ represent the features of the three different encodings while using the corresponding branches of the network. $seq_{1}$, $seq_{2}$, and $seq_{3}$ show the input representations from the three encoding schemes, respectively, whereas f1, f2, and f3 show the three projection branches of the network. In the third step, the features obtained from the second step are concatenated to form a joint latent representation as follows:

(7) $$z_{f}=concat\left(\left[z_{1},\;z_{2},\;z_{3}\right]\right),$$

where $z_{f}$ represents the final concatenated features from the three branches of the network. In the fourth step, the concatenated features are classified using two fully connected layers of the bCNN-Methylpred network as follows:

(8) $$y=fc\left(z_{f}\right),$$

where y represents the m6A sites in the RNA sequence, and fc shows the function of the two fully connected layers.

To optimize the parameters of the proposed models, we used the binary cross-entropy loss function, as follows:

(9) $$Loss=-\frac{1}{N}\overset{N}{\underset{i=1}{\sum }}\left(y_{i}\cdot \log\left(p\left(y_{i}\right)\right)+\left(1-y_{i}\right)\cdot \log\left(1-p\left(y_{i}\right)\right)\right),$$

where $y_{i}$ denotes the corresponding label of sample i; $p\left(y_{i}\right)$ denotes the output of the network; N is the total number of samples.

### 3.3. Benchmark Datasets

To analyze the performance of the proposed network by predicting the m6A sites in the RNA sequences, we first selected four different species benchmark datasets, followed by miCLIP-Seq benchmark datasets.

#### 3.3.1. Four Benchmark Datasets

The datasets used in this study included the *H. sapiens*, *M. musculus*, *S. cerevisiae*, and *A. thaliana* datasets. All sequences of these four benchmark datasets contained A at the center. The positive sequences were sequences centered with true m6A sites, whereas the negative sequences were without m6A sites. The *H. sapiens* benchmark dataset was generated by the authors of [38]. This dataset comprised 1130 positive sequences and 1130 negative sequences, where the length of each sequence was 41nt. In [22], the authors prepared an *M. musculus* benchmark dataset. The length of each sequence in this dataset was 41nt as well. The *M. musculus* benchmark dataset comprised 725 positive sequences and 725 negative sequences. The *S. cerevisiae* benchmark dataset was developed by the authors of [39]. This dataset contained 1307 positive sequences and 1307 negative sequences. The length of each sequence was 51nt. The *A. thaliana* benchmark dataset was created by the authors of [40]. It contained 2100 positive sequences and 2100 negative sequences, where the length of each sequence was 101 nt. A summary of these benchmark datasets is presented in Table 2. We used k-fold cross-validation to evaluate the performance of the proposed approach. According to a recent literature survey, the evaluation of the model using k-fold cross-validation or the jackknife test does not require a dedicated testing dataset. The different k-fold outcomes can be considered as different independent test datasets.

#### 3.3.2. miCLIP-Seq Datasets

Next, we selected the miCLIP-Seq dataset that can identify m6A sites at a single-base resolution. We obtained this dataset from the same source as the SRAMP for *humans and mice* [41,42], which included five cell lines and tissue types, i.e., A549, CD8T, HEK293, brain, and liver. To generate positive and negative samples, we performed the procedure detailed in [43]. For the positive samples, we defined the sequences with a window measuring 101 nt that contained m6A sites. We first mapped the m6A sites to the longest transcript of the gene using the ENSEMBL database (http://www.ensembl.org/, accessed on 27 July 2021) and then randomly located the m6A sites in the fixed-size windows. Subsequently, we extracted the surrounding sequence with a length of 101 nt. Because m6A sites have been reported to agglomerate [21], to avoid sample redundancy before locating the m6A sites randomly, we first merged them within 50 nt and selected the centered one among the merged sites. To generate negative samples, we selected the length near the window that did not contain m6A sites. These windows were generated by a stride of 10nt and 100 steps, and the corresponding negative sample closest to the positive sample was obtained. For the two closest samples on both sides of the positive sample, we selected one sample randomly. Further descriptions of the datasets are shown in Table 3.

### 3.4. Performance Evaluation

To evaluate the effectiveness of the proposed approach in terms of the identification of m6A sites in RNA sequences, we performed an extensive comparative quantitative analysis using the six abovementioned different benchmark datasets. For performance evaluation, the sequences were first generated using the approaches discussed in Section 3.3.1 and Section 3.3.2. Once the positive and negative sequences were generated, we encoded them in a format acceptable for the model, i.e., in the binary format using the three encoding schemes discussed above (i.e., circular encoding, one-hot encoding, and NCP encoding). Subsequently, they were used as an input to the proposed network. The proposed network comprises three branches, where the first branch uses the sequence encoded by the circular encoding, the second branch uses the sequence encoded by the one-hot encoding scheme, and the last branch uses the NCP encoded sequence. A detailed description of the three RNA sequence encoding schemes is provided in Section 3.1. After encoding the RNA sequences, we independently trained the proposed network on the individual species and predicted the modification sites in the specified species.

#### 3.4.1. Performance Evaluation Metrics

We trained the proposed network using the four abovementioned different benchmark datasets using 10-fold cross-validation techniques for m6A site predictions. These four benchmark datasets were segregated into 10 mutually exclusive folds. Among them, one fold was used for testing, one fold for validation, and the remaining for training the proposed network. The training was performed recursively. The final results of the proposed model were obtained by averaging the results from all the folds. Subsequently, we performed a comparative quantitative analysis based on the following metrics: accuracy, sensitivity, specificity, Mathew’s correlation coefficient (MCC), F1-score, and area under the curve (AUC). These metrics are defined individually as follows:

Accuracy presents the overall evaluation of the model in terms of correctly detected positive and negative samples. It is expressed as follows:

(10) $$Accuracy=\frac{Tp+Tn}{Tp+Tn+Fp+Fn},$$

where Tp represents true positive. True positives show accurately detected positive class samples. Tn represents true negative. True negatives show accurately detected negative class samples. Fn represents false negative. False negatives imply that the samples belonging to the positive class are incorrectly classified as negative. Similarly, Fp represents false positive. False positives imply that the samples belonging to the negative class are incorrectly classified as positive.

Sensitivity reflects the proportion of true-positive classes that are correctly identified, expressed as follows:

(11) $$Sensitivity=\frac{Tp}{Tp+Fn}$$

Specificity measures the proportion of true-negatives classes that are correctly identified, expressed as follows:

(12) $$Specificity=\frac{Tn}{Tn+Fp}$$

MCC reflects the performance of the classification model and is expressed as follows:

(13) $$MCC=\frac{Tp\times Tn-Fp\times Fn}{\sqrt{\left(Tp+Fp\right)\left(Tp+Fn\right)\left(Tn+Fp\right)\left(Tn+Fn\right)}}$$

The F1-score is a measure of test accuracy. It is defined as:

(14) $$F1\;\mathrm{score}=\frac{Tp}{Tp+\frac{1}{2}\left(Fp+Fn\right)}$$

The AUC is a performance measurement for classification models. It indicates the degree or measure of separability between different classes. Specifically, the AUC measures the separating or distinguishing capability of a model between different classes. In terms of classifying different classes, the higher the AUC, the better the model.

#### 3.4.2. Prediction on Four Benchmark Datasets

Different experiments were performed to evaluate the effectiveness of the proposed network. In the first experiment, we analyzed the proposed network by directly concatenating the input representations from the three encoding schemes; subsequently, we input the concatenated input to a single branch CNN to predict the m6A sites. In this step, we first concatenated all three encodings and then used the concatenated encodings as inputs to the network. Subsequently, we used the proposed three-branch CNN, which used the three input representations separately in their corresponding branches, to predict the m6A sites in the RNA sequences. In terms of the three branches’ CNN, the input representation was not concatenated in the input, but the features of each input were concatenated in the feature space of the network. It is noteworthy that the CNN transformed the nonlinear input space into a linear feature space. Hence, we concatenated the linear features in the feature space of the corresponding branch and used the concatenated features to detect m6A sites. The results generated by the concatenations in the input space and feature space for all four species are listed in Table 4. Based on our analysis, we observed that the three-branch CNN predicted m6A sites more accurately than the single-branch CNN. This validates that concatenating features rather than input directly resulted in a more robust and accurate model. In addition to performing concatenations in the input space and feature space, we evaluated the effectiveness of all three features in different combinations. In the first combination, we combined the features generated from one-hot encoding and the NCP. Subsequently, we evaluated the performance of the model in terms of different evaluation metrics, as listed in Table 5. In the second combination, we used one-hot encoding with circular encoding. Finally, in the third combination, we used circular encoding and NCP combinations. We observed that the combination of circular encoding with one-hot and NCP indicated slight improvements in terms of accuracy, sensitivity, specificity, MCC, and AUC, as listed in Table 5. Subsequently, we evaluated the effects of the individual encoding schemes on m6A site prediction in the RNA sequence of *M. musculus* species, as listed in Table 6. The effect of one-hot and NCP encoding schemes on the prediction of RNA sequence modification was evaluated previously using pm6A-CNN [36]. However, to demonstrate the effect of circular encoding, we performed a comparative quantitative analysis among the individual encoding schemes for the prediction of RNA sequence modification. We observed that circular encoding improved the accuracy, sensitivity, and MCC compared with one-hot encoding and NCP, as listed in [36].

Next, we performed a comparative quantitative analysis of the proposed approach with existing state-of-the-art approaches such as M6AMRFS [31], iN6-Methyl [35], iMRM [44], and pm6A-CNN [36] using H. *sapiens* and *M. musculus* species. Results of the analysis are shown in Table 7 and Table 8. Considering the quantitative analysis in terms of the *H. sapiens* species (shown in Table 7), we observed that the proposed approach outperformed M6AMRFS by 3.12%, 10.92%, and 5.02% in terms of accuracy, sensitivity, and MCC, respectively. The accuracy, sensitivity, MCC, and AUC of the proposed approach were 3.02%, 14.12%, 4.82%, and 7.6% higher than those of iN6-Methyl, respectively, and 3.12%, 10.52%, 6.32%, and 3.9% higher than those of iMRM, respectively. Similarly, the accuracy, sensitivity, MCC, and AUC of the proposed approach were 0.52%, 4.32%, 0.52%, and 1.9%, higher than those of pm6A-CNN, respectively. Similarly, considering the *M. musculus* species, the proposed approach indicated improved performances compared with all the competitors listed in Table 8 in terms of accuracy, sensitivity, and AUC. Its specificity was higher than those of the other approaches for both species. Sensitivity indicates the correct detection of true-positive classes, whereas specificity indicates the correct detection of true-negative classes. The higher the sensitivity and specificity, the better is the performance of the model. We observed that the specificities of the other approaches were higher than that of the proposed approach, but the sensitivities were significantly lower. This is not a good indicator of the overall accuracy of the models. By contrast, the proposed approach demonstrated high sensitivity and specificity, and the difference between them was insignificant; hence, the overall accuracy was high.

In terms of the *S. cerevisiae* species, we performed a quantitative analysis of the BERMP [45] competitor alongside the competitors listed in Table 7 and Table 8. The quantitative analysis results are shown in Table 9. In this analysis, we observed that the proposed approach outperformed BERMP, M6AMRFS, iN6-Methyl, iMRM, and pm6A-CNN in terms of accuracy by 20.28%, 14.67%, 13.57%, 11.17%, and 3.87%, respectively. Regarding the other metrics, including sensitivity, MCC, and AUC, the proposed approach yielded higher values than the listed competitors. The specificity of BERMP was slightly higher than that of the proposed approach, whereas the values of other metrics of BERMP such as sensitivity and MCC were extremely low. The specificity for the remaining competitors was lower than that of the proposed approach.

**Figure 3:**
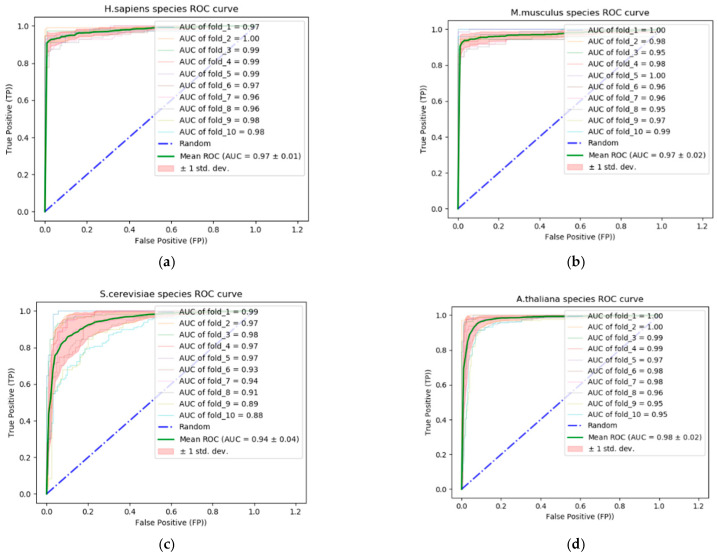
auROC curve of the proposed approach for four different benchmark datasets: *H. sapiens*, *M. musculus*, *S. cerevisiae*, and *A. thaliana*. (**a**,**b**) show auROC curves of the proposed approach for *H. sapiens* and *M. musculus* species. (**c**,**d**) show auROC curves for *S. cerevisiae* and *A. thaliana* species.

In addition to the competitors listed in Table 7, Table 8 and Table 9, we included an additional competitor, RFAthM6A [40], for the comparative quantitative analysis of the *A. thaliana* species. The quantitative analysis results are shown in Table 10. This analysis shows that the proposed approach outperformed BERMP, M6AMRFS, M6AMRFS, and pm6A-CNN in terms of accuracy, sensitivity, specificity, MCC, and AUC. Species A. *thaliana* was not included in iN6-Methyl and iMRM for the quantitative analysis. The quantitative results of the proposed network in terms of accuracy, sensitivity, specificity, and MCC for all benchmark datasets indicated the robustness of the proposed approach in terms of m6A site identification using a specific branch structure and the novel circular encoding scheme. Figure 3 shows the AUC and receiver operating characteristics (ROC) of the proposed approach in addition to the standard deviation in 10 folds using the four benchmark datasets: H. *sapiens*, *M. musculus*, *S. cerevisiae*, and *A. thaliana*. The AUC–ROC (or auROC) curve is a performance measurement of the proposed approach for m6A site identification. ROC is a probability curve, whereas AUC represents the degree or measure of separability. This metric evaluates the model in terms of correctly classified positive and negative classes. The higher the AUC, the better is the prediction of the positive and negative classes.

#### 3.4.3. Prediction on miCLIP-Seq Datasets

In addition to the abovementioned benchmark datasets, i.e., *H. sapiens*, *M. musculus*, *S. cerevisiae*, and *A. thaliana*, we evaluated our model on a mammalian dataset that comprised *the human and mouse* miCLIP-seq dataset. We compared our model with DeepM6ASeq [43] and other classifiers including random forest, logistic regression, and support vector machine, which were mentioned in [43]. The overall comparative analysis in terms of accuracy, F1-score, MCC, and AUC are listed in Table 11. The approaches listed in Table 11 used the combined testing samples of *human and mouse* species for testing, whereas we tested our model on the combined as well as individual species. Based on this analysis, we observed that the proposed model outperformed DeepM6ASeq [43] and other classifiers mentioned in [43] in terms of accuracy, F1-score, MCC, and AUC.

### 3.5. Discussion

Predicting the modification sites in different RNA sequences is crucial as it enables a better understanding of their functional mechanisms in different biological processes. This prediction involves three main factors: accuracy, rapidity/real-time verification, and simplicity in terms of designing verification methods. Based on these factors, modification sites in different RNA sequences can be predicted via two main approaches. One is an experiment-based modification site prediction, and the other is ML- and DL-based modification site prediction. Experiment-based methods for modification site identification are accurate but extremely slow and laborious. By contrast, ML- and DL-based approaches are key approaches for replacing experiment-based approaches, owing to their rapid verification and simplicity; however, accuracy is a major concern. Although existing ML- and DL-based approaches attempt to achieve the desired accuracy, their inherent limitations prevent them from achieving the desired goal. For example, most of these approaches rely on input representations and combine them to achieve the desired goal. If the input representation is linear, then a sufficient and acceptable accuracy level can be achieved. However, in terms of highly nonlinear input representations, they fail to perform accurately. Considering these two issues, we performed two main modifications: introducing a circular encoding scheme and obtaining linear representations from nonlinear representations. In circular encoding, a pair of nucleotide bases is used in every possible configuration and a binary code is assigned to the designated pair. This encoding scheme creates a linear representation of an RNA sequence. Similarly, the CNN clusters feature from a specified input and segments these clusters in its feature space. More explicitly, it converts the nonlinear representation into a well-separated linear space. Hence, we first used the three input representations as inputs to the three separate designated branches. Each of these branches converts input representations into their corresponding linear feature spaces. Once the input representation is converted to its linear representation, we combine the feature space from each branch into a single feature. Next, we input it to a fully connected classification layer, which is then classified into their corresponding modification sites. The proposed approach ensures high accuracy, as well as rapid verification and simplicity. The comparative analysis of the proposed approach with machine learning approaches is described in Table 12. The parameters used in the proposed approach and the competitor methods are listed in Table 13. These parameters include the filter arrangement in the convolutional layers, filter size, strides, dropout, and neurons used in the fully connected layers. In terms of the proposed approach and iN6-Methyl [35], two convolutional layers and two fully connected layers were involved, whereas the pm6A-CNN [36] contained four convolutional layers and five fully connected layers. For the proposed approach, the total number of filters was less than that of the competitors.

### 3.6. Deep Learning Models

A deep neural network typically comprises convolutional layers followed by fully connected layers. Convolutional layers serve two main purposes in deep neural networks. First, they can easily manage high-dimensional inputs. Second, they preserve the fine details of the inputs in the form of convolutional filters. The convolution layers project the high-dimensional input to a lower-dimensional space, which can be managed easily by the fully connected layers. Subsequently, the fully connected layers classify or categorize the input data into their corresponding classes in terms of classification. This entire neural network can be considered as a function approximator, which uses input x and generates the corresponding label y, as follows:

(15) y=f(x)

The architecture of a deep neural network is analogous to that of the neuronal connectivity arrangement in the human brain and is inspired by the visual cortex organization. Individual neurons respond to stimuli only in a limited region of the visual field, known as the receptive field. A group of such fields overlaps to encompass the entire visual area. Furthermore, the deep neural network comprises many layers and can learn complex structures where shallow classical ML approaches fail. Most primitive ML approaches are hand-engineered, but CNNs learn the features automatically. In addition, parameter tuning is laborious in ML approaches because they lack generalization. As the CNN is a more generalized approach than traditional ML approaches, its accuracy, F1-score, MCC, and AUC are higher than those of traditional ML approaches, including logistic regression, random forest, and support vector machine, as shown in Table 12.

### 3.7. Biological Interpretation of Proposed Model

In many biological applications, researchers are more interested in biological mechanisms rather than the prediction accuracy of a predictive model [46,47]. For example, the main motivation for building accurate DL models to predict m6A sites in RNA sequences is to investigate them from a biological perspective. This investigation can be performed by interpreting the trained model. Although DL models achieve high accuracy, they are highly nonlinear, and interpreting them is more challenging than interpreting standard statistical models. To demonstrate the capability of the proposed model by interpreting them, we performed a well-established interpretation procedure, i.e., in silico mutagenesis [48,49,50]. In this procedure, we mutated individual nucleotide bases, A, C, G, and U, sequentially and individually while preserving the remaining nucleotide bases and tracked the model behavior using the mutated sequence. Although this approach is computationally expensive, it is simple and meaningful. Using this approach, we first generated the output using the reference sequence; subsequently, we generated the output of the corresponding mutated sequence. Next, we obtained the absolute difference between the output generated from the reference sequence and the mutated sequence and then stored the results of the mutated sequence in addition to the absolute difference. To determine the model behavior in terms of the entire dataset, we combined the individual generated predictive scores and then calculated the average.

**Figure 4:**
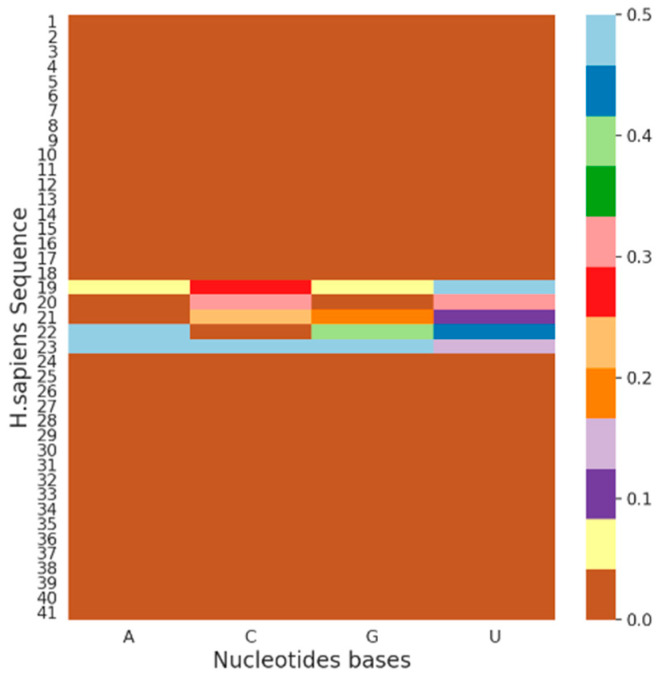
Visualization of mutation effect using in silico mutagenesis approach for *H. sapiens* species. This shows the difference between the mutated sequence and reference. Nucleotide base A in the sequence was not affected significantly compared with other nucleotides bases of *H. sapiens* species shown in the figure.

To visualize the effects of the model on the mutated sequences for each feature, we generated a heatmap. This is enabled by the capability of the DL model in visualizing each convolution kernel as a heatmap. We generated a heatmap of the mutated sequence and the absolute difference for the *H. sapiens* species, as shown in Figure 4. We intentionally mutated the sequences individually for all the nucleotide bases and then generated the output to determine the behavior of the model toward the mutated sequence. However, we discovered that it was difficult to interpret and analyze the effect of modification visually because the values of the generated heatmap were low. For a more meaningful representation, we generated heatmaps for the absolute difference between the reference and mutated sequence outputs. Figure 4 shows the heatmaps from the absolute difference for the *H. sapiens* species. The color bar shows the effect of mutation of a particular nucleotide base in a sequence. For example, the brown color value is 0 and indicates the least effect, whereas the light blue color shows the most prominent effect while mutating a particular nucleotide base in a sequence. Considering the methyladenosine modification, we observed that the mutation effect was greater at the center of the sequence. In addition, this analysis indicates that the model can better reflect the modifications of nucleotide bases C, G, and U than that of nucleotide base A because modifications have already occurred in the latter nucleotide base. Furthermore, through the alteration procedure of different nucleotide bases in a specified sequence, the capability of the model in identifying other modifications in the genome can be verified by considering other nucleotide bases.

## 4. Conclusions

In this study, we developed an accurate and robust branch CNN that can effectively identify modification sites in different RNA sequences using four benchmark datasets, i.e., *H. sapiens*, *M. musculus*, *S. cerevisiae*, and *A. thaliana*. The proposed network uses three different input representations in their corresponding designated branches. These input representations were transformed into feature spaces, which were subsequently concatenated in the feature space. Next, the concatenated feature space was used to predict the modification sites in different RNA sequences. In addition to one-hot encoding and NCP, we proposed a new circular encoding scheme that encodes every possible combination of the input sequence. The circular encoding scheme further improves the accuracy of the proposed approach. To validate the effectiveness of the proposed approach, we performed a comparative quantitative analysis using the aforementioned benchmark datasets. It was discovered that the proposed approach outperformed state-of-the-art methods, as shown in Table 6, Table 7, Table 8, Table 9, Table 10, Table 11, Table 12 and Table 13.

## Figures and Tables

**Table 1:** Hyperparameter optimization of bCNN-Methylpred.

Hyperparameters	Choices
L2 Kernel Regularization	1 × 10^−3^, 1 × 10^−3^, 1 × 10^−3^
L2 bias Regularization	1 × 10^−4^, 1 × 10^−4^, 1 × 10^−4^
Group Normalization	4, 4, 4
Drop-out	0.5, 0.5, 0.5
Filters	(32, 16) (32, 16) (32, 16)
FC Neurons	(24, 1), (24, 1), (24, 1)
Learning rate	0.001
Learning rate reduction factor	0.01

**Table 2:** Details pertaining to four species of benchmark datasets.

Scheme	Positive Sequences	Negative Sequences	Total Samples	Sequence Length
*H. sapiens*	1130	1130	2260	41 nt
*M. musculus*	725	725	1450	41 nt
*S. cerevisiae*	1307	1307	2614	51 nt
*A. thaliana*	2100	2100	4200	101 nt

**Table 3:** Details pertaining to miCLIP-Seq datasets.

Scheme	Training Samples	Testing Samples	Validation Samples	Total Samples	Sequence length
*Human*	36,998	12,331	12,332	61,661	101 nt
*Mouse*	28,271	9422	9424	47,117	101 nt

**Table 4:** Comparison of m6A site prediction using concatenations in input space and feature space using four species of benchmark datasets.

Species	Concatenation	Accuracy	Sensitivity	Specificity	MCC
*H. sapiens*	Concatenation in input space Concatenation in feature space	0.9341 0.9491	0.8858 0.9274	0.9823 0.9708	0.8732 0.8996
*M. musculus*	Concatenation in input space	0.9317 0.9428	0.8896 0.9256	0.9737 0.9610	0.8668 0.8865
	Concatenation in feature space				
*S. cerevisiae*	Concatenation in input space Concatenation in feature space	0.8570 0.8846	0.8623 0.8754	0.8518 0.8937	0.7154 0.7701
*A. thaliana*	Concatenation in input space Concatenation in feature space	0.9333 0.9480	0.9400 0.9457	0.9267 0.9505	0.8673 0.8963

**Table 5:** Evaluation of effectiveness of different feature combinations using *M. musculus* species.

Approaches	Accuracy	Sensitivity	Specificity	MCC	AUC
One-hot+NCP	0.9496	0.9366	0.9626	0.9007	0.9726
One-hot+Circular	0.9498	0.9353	0.9642	0.9012	0.9794
Circular+NCP	0.9538	0.9380	0.9696	0.9093	0.9788

**Table 6:** Comparison of different encoding schemes using *M. musculus* species.

Approaches	Accuracy	Sensitivity	Specificity	MCC
One-hot	0.935	0.893	0.976	0.874
NCP	0.928	0.895	0.961	0.859
Circular	0.942	0.920	0.965	0.887

**Table 7:** Comparison of m6A site prediction using proposed and state-of-the-art approaches based on *H. sapiens* species.

Approaches	Accuracy	Sensitivity	Specificity	MCC	AUC
M6AMRFS [31]	0.910	0.820	1.000	0.833	-
iN6-Methyl [35]	0.911	0.788	1.000	0.835	0.903
iMRM [44]	0.910	0.824	0.995	0.820	0.940
pm6A-CNN [36]	0.936	0.886	0.986	0.878	0.960
bCNN-Methylpred	0.941	0.929	0.953	0.883	0.979

**Table 8:** Comparison of m6A site prediction using proposed and state-of-the-art approaches based on *M. musculus* species.

Approaches.	Accuracy	Sensitivity	Specificity	MCC	AUC
M6AMRFS [31]	0.793	0.898	0.828	0.758	-
iN6-Methyl [35]	0.895	0.821	1.000	0.807	0.913
iMRM [44]	0.889	0.783	0.995	0.779	0.820
pm6A-CNN [36]	0.938	0.904	0.972	0.88	0.970
bCNN-Methylpred	0.945	0.937	0.953	0.891	0.942

**Table 9:** Comparison of m6A site prediction using proposed and state-of-the-art approaches based on *S. cerevisiae* species.

Approaches	Accuracy	Sensitivity	Specificity	MCC	AUC
BERMP [45]	0.686	0.471	0.901	0.412	0.800
M6AMRFS [31]	0.742	0.752	0.733	0.480	-
iN6-Methyl [35]	0.753	0.761	0.746	0.507	0.803
iMRM [44]	0.777	0.770	0.780	0.555	0.850
pm6A-CNN [36]	0.850	0.846	0.855	0.703	0.920
bCNN-Methylpred	0.889	0.890	0.888	0.779	0.943

**Table 10:** Comparison of m6A site prediction using proposed and state-of-the-art approaches based on *A. thaliana* species.

Approaches	Accuracy	Sensitivity	Specificity	MCC	AUC
BERMP [45]	0.860	0.818	0.901	0.722	0.927
M6AMRFS [31]	0.854	0.873	0.835	0.709	-
RFAthM6A [40]	0.810	0.806	0.814	0.621	0.926
iN6-Methyl [35]	-	-	-	-	-
iMRM [44]	-	-	-	-	-
pm6A-CNN [36]	0.925	0.923	0.926	0.850	0.970
bCNN-Methylpred	0.942	0.944	0.941	0.885	0.977

**Table 11:** Comparison of m6A site prediction using proposed and the state-of-the-art approaches based on *human and mouse* species.

Approaches	Species	Accuracy	F1-score	MCC	AUC
DeepM6ASeq [43]	*Human*+*Mouse*	0.764	0.762	0.528	0.844
Random forest	*Human*+*Mouse*	0.747	0.756	0.494	0.826
Logistic regression	*Human*+*Mouse*	0.743	0.736	0.487	0.824
Support vector machine	*Human*+*Mouse*	0.736	0.732	0.472	0.818
bCNN-Methylpred	*Human*+*Mouse*	0.801	0.809	0.604	0.876
bCNN-Methylpred	*Human*	0.825	0.828	0.652	0.892
bCNN-Methylpred	*Mouse*	0.824	0.826	0.648	0.896

**Table 12:** Comparison of m6A site prediction using proposed CNN-based approach and state-of-the-art machine learning approaches based on *human and mouse* species.

Approaches	Species	Accuracy	F1-Score	MCC	AUC
bCNN-Methylpred (CNN)	*Human*+*Mouse*	0.801	0.809	0.604	0.876
Logistic regression (ML)	*Human*+*Mouse*	0.743	0.736	0.487	0.824
Random forest (ML)	*Human*+*Mouse*	0.747	0.756	0.494	0.826
Support vector machine (ML)	*Human*+*Mouse*	0.736	0.732	0.472	0.818

**Table 13:** Parameter comparison of proposed approach with competitor methods.

Hyperparameters	bCNN-Methylpred	iN6-Methyl (34)	pm6A-CNN (35)
Filters Arrangements in Convolutional Layers	(32,16)	(32,32)	(5,8,10,16)
Filter size	(5,3)	(5,5)	(3,5,7)
Stride	(1,1)	(1,2)	(2,3,4)
Drop-out	0.5, 0.5, 0.5	0.2, 0.2	0.2, 0.3, 0.4, 0.5
FC Neurons	(24,1)	(128,1)	(1,5,8,10,16)
